# T-Scan Novus System in the Management of Splints—Pilot Study

**DOI:** 10.1055/s-0041-1736315

**Published:** 2021-12-04

**Authors:** Tanya Bozhkova, Dobromira Shopova

**Affiliations:** 1 Department of Prosthetic Dentistry, Faculty of Dental Medicine, Medical University, Plovdiv, Bulgaria

**Keywords:** 3Shape, splint, bruxism, digital splint design, T-Scan Novus system

## Abstract

The purpose of this pilot study was to demonstrate the capabilities of the T-Scan Novus system in bruxism treatment by splints. Bruxism patients underwent treatment with a splint made by additive manufacturing. Intraoral scanning was performed using Trios Color (3Shape), and digital design was performed using 3Shape Dental system design - splint studio. The biocompatible material Dental LT Clear Resin was printed using a Formlabs Form 2 printer. The T-Scan Novus system with a software attached to it, version 9.1, was used for digital examination of the occlusion. A splint with an occlusal thickness of 2.5 mm was developed and software adapted with relief to antagonists. The digitally set occlusion with even contacts turned out to be clinically unbalanced. After adjusting with T-Scan Novus, a balanced occlusion was achieved in the right and left halves. The treatment of bruxism with splint therapy continues to be the main method. Its combination with digital technologies allows more precise constructions and more accurate balancing of occlusal relationships.

## Introduction

The causes of bruxism can be changes in occlusion (due to prosthetic, orthodontic or therapeutic treatment or periodontal disease), altered neuromuscular programming (due to inflammatory diseases of the temporomandibular joint [TMJ], muscles, myofascial pain), and changes in intrinsic and extrinsic factors (systematic disease, psychosomatic disease, psychosocial factors). The causes of bruxism deepen over time and can be defined as its clinical manifestation—abraded teeth, occlusal trauma, TMJ disorders, and dysfunction in masticatory muscles. 1

The methods of treatment include occlusal therapy by different methods—selective grinding, composite restorations, prosthetic restorations, and orthodontic treatment. Proper therapy in all requires stabilization of distal contacts. A variant of prosthetic treatment by removable constructions is with the help of an occlusal splint. Splint treatment options include two main types, according to Okeson, stabilization splint and repositioning splint. 2 Four additional types of splints with specific indications are described as follows: pivot splint, soft splint, anterior bite splint, and posterior bite splint. 3 4 Bumann added terms that combine different therapeutic methods—relation splint whose purpose is to normalize the activity of the muscles of mastication by equalizing posterior tooth contacts; decompression splint, which should be made to fit the centric jaw relation existing at the moment (“momentary centric”); and verticalization splint, which increased vertical dimension of occlusion (VDO). 1 The splints' aims are to redistribute the occlusal forces and reduce the activity of the masticatory muscles. 5 To achieve a therapeutic effect, it is necessary to wear it almost all the time. 6 7 8

In recent years, modern methods of occlusal splints' creation using CAD/CAM technology and additive manufacturing have been increasingly developed. With the progress of digital technology, the design, production, and manufacture of splints can be further developed. Berntsen et al compared the occlusion produced by intraoral scanning and addition with traditional methods and found that it is statistically significant. 9 Salmi et al used a scanner to receive the models. The upper and lower jaw model is imported into CAD digital design software but does not effectively improve occlusion. When the occlusal splint is placed in the patient's mouth, the posterior teeth are still in contact and the anterior teeth are slightly open. Based on the above problems, this research group used internal scanning to obtain occlusal connection data after upper and lower jaw augmentation and also utilized CAD software to provide higher VDO. After occlusion, the teeth of the upper and lower jaw can still be touched evenly to achieve the patient's occlusion. 10 Stabilizing and repositioning splints are the main types described by Okeson, 2 and it is digitally possible to combine different elements of both to obtain an individual approach for the patient. 11 An occlusal splint, planned digitally with virtual mandibular repositioning software, seems to be able to achieve the position that has been established by the operator in subjects with TMD. 12

The purpose of this pilot study is to demonstrate the capabilities of the T-Scan Novus system in the treatment of bruxism with occlusion splints.

## Case Report

This report was approved by the Ethics Commission of the Medical University - Plovdiv. Each patient signed an informed consent form. It included 15 patients with symptoms of bruxism, selected according to the following criteria.

Criteria for inclusion in the study are as follows:

- Age from 18 to 25 years.
- Patients after orthodontic treatment in the retention phase (instead a retainer).
- Anamnesis of bruxism—pressing or clenching of teeth, pain in the TMJ, muscle discomfort (in most of the cases all of them).
- Presence of clinical symptoms of bruxism—exostoses, teeth with attritions, muscle hyperactivity, cracking TMJ (in most of the cases all of them).

To make the occlusal splints, patients were scanned with an intraoral Trios Color scanner (3Shape), and the digital impressions were emailed to the dental laboratory, in order to design the occlusal splint. Software 3Shape Dental system design - splint studio was used. The finished design was printed using a Formlabs Form 2 printer from the biocompatible material Dental LT Clear Resin.

The T-Scan Novus system was used for occlusal splint adjustment, which consists of a handle, sensor holder, sensor, and software version 9.1.

Technique for registration of occlusal contacts—patients are placed in a sitting position with straight back and head, the horizontal plane parallel to the floor. After undercutting a suitable size sensor, it is placed in the handle. The device is placed in the patient's mouth with the index finger of the holder between the upper central incisors. To record occlusal contacts, patients were asked to close the maximum intercuspation position (MIP) for 2 to 4 seconds and then to disarticulate the lower jaw by moving it forward or to the sides (before excursion movement). The system software automatically calculates the registered data and displays it on the computer screen in real time.

The following parameters were set for the creation of the splint:

- Vestibular splint thickness 1 mm.
- Protrusion to class I by angle 1 mm.
- Opening 2.5 mm.
- Medium/later protrusion 0 mm.
- Raise surface—a relief to antagonists.

## Results

When placed in the patient's mouth, the splint was very precisely adapted to the tooth surfaces. However, a difference with the digitally designed one was conclusively established. To more accurately identify the differences, the T-Scan Novus system was used, and the contacts were marked clinically with 40 µ articulation paper.

To illustrate the proposed method for splint adjustment with the T-Scan Novus system, we randomly selected one of the patients included in the study.

**Fig. 1:**
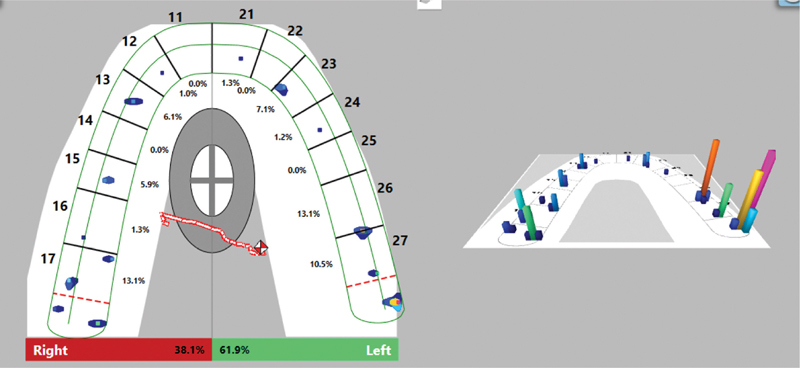
Occlusal contacts in the maximum intercuspation position (MIP) during the initial placement of the splint.

**Fig. 2:**
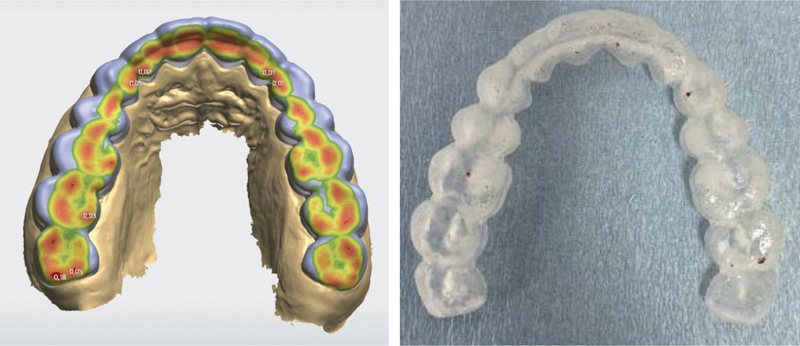
Digital design and registered occlusal contacts with articulation paper 40 µ at the initial placement of the splint.

The registered occlusal contacts were illustrated as two- and three-dimensional images of different colors, according to the applied force in them. During the initial placement of the splint, uneven contacts are established in single teeth. The center of forces (COF) is strongly shifted to the left and is out of the COF target. The forces in the two halves of the dentition are not evenly distributed, as it is 38.1% in the right half and 61.9%in the left. The left half of the dentition bears almost double the load, which would affect the activity of the masticatory muscles and the TMJ. With articulation paper 40 µ, we registered the contacts in the patient mouth. Figs. 1 and 2 show the occlusal contacts during the initial placement of the splint.

In the analysis of the occlusal film, after the occlusal adjustment of the splint, evenly distributed contacts were established in the two halves of the dentition—46.7% on the right and 53.3% on the left. The trajectory of the COF is shorter and is in the center of COF. When registering contacts with articulation paper, larger and smaller markings are found.

**Fig. 3:**
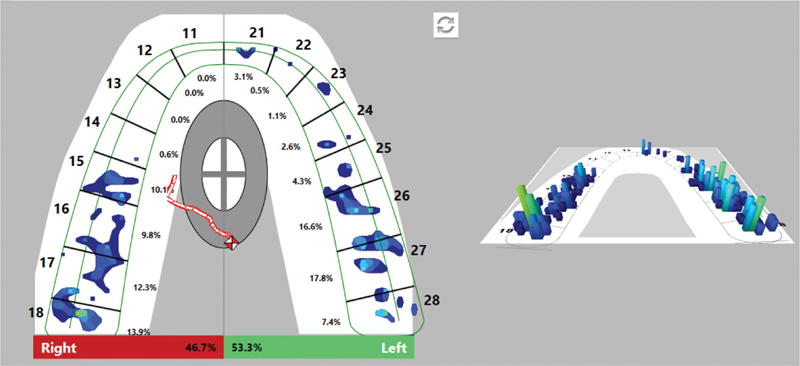
Occlusal contacts in the maximum intercuspation position (MIP) after adjustment.

**Fig. 4:**
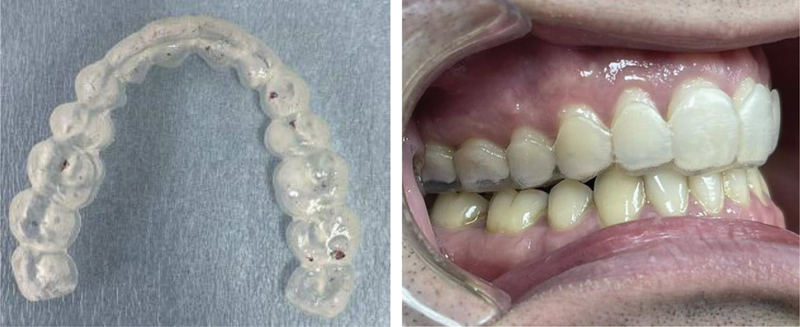
Registered occlusal contacts with articulation paper 40 µ after occlusal adjustment and clinical view of the patient.

Figs. 3 and 4 show the distribution of occlusal contacts in the MIP after occlusal adjustment.

## Discussion

When registering occlusal contacts with articulation paper only, COF and distribution of forces cannot be assessed. Previous research has shown that articulation paper is unable to record the forces of occlusal contacts and time, making it an inadequate indicator of concurrent occlusal contact. 13 When registering contacts with articulation paper, larger and smaller markings can be found, which would confuse which of the contacts are strong or weak, determined only by their intensity and size. 14 The T-Scan system not only measures the forces and time of each contact but also converts this information into easily interpreted graphs. Over the years, it has proven its usefulness during occlusal analysis in several studies. 15 16 17 18 The correlation of forces in occlusion in MIP between the subjective interpretation of the dentist, the patient's perception and the T-Scan system were studied, and it was proved that when using T-Scan as a gold standard, the subjective interpretation of the doctor is more clinically reliable than the patient's perception. 19 Karakis performed a study with the T-Scan system synchronized with BioEMG III in patients with bruxism before and after splint treatment. 20 When the occlusal splint is placed, increased salivation is reflexively detected. The only occlusal indicator that is not affected by saliva is the T-Scan system. 21

## Conclusion

The present study found that the design of the splints did not produce uniform occlusal contacts, which was proven with the T-Scan Novus system. Тhe influence of balanced occlusion in bruxism through the system brings the splint therapy closer to a successful outcome. These results will be tested in future studies with a larger number of patients. To achieve balanced occlusal ratios in the treatment of splint bruxism, we recommend the usage of the T-Scan system for occlusal adjustment.
